# Hepatocyte apoptosis is tumor promoting in murine nonalcoholic steatohepatitis

**DOI:** 10.1038/s41419-020-2283-9

**Published:** 2020-02-03

**Authors:** Petra Hirsova, Friederike Bohm, Ester Dohnalkova, Barbora Nozickova, Mathias Heikenwalder, Gregory J. Gores, Achim Weber

**Affiliations:** 1 Division of Gastroenterology and Hepatology, Mayo Clinic, Rochester, MN USA; 2 Institute of Clinical Biochemistry and Diagnostics, University Hospital Hradec Kralove, Hradec Kralove, Czech Republic; 3 Department of Pathology and Molecular Pathology, University and University Hospital Zurich, Zurich, Switzerland; 4 Department of Chemistry and Biochemistry, Mendel University in Brno, Brno, Czech Republic; 5 Division of Chronic Inflammation and Cancer, German Cancer Research Center (DKFZ), Heidelberg, Germany; 6 Institute of Molecular Cancer Research (IMCR), University Zurich, Zurich, Switzerland

**Keywords:** Liver cancer, Chronic inflammation

## Abstract

Nonalcoholic fatty liver disease is the most common chronic liver disease and may progress to nonalcoholic steatohepatitis (NASH) and hepatocellular carcinoma (HCC). The molecular determinants of this pathogenic progression, however, remain largely undefined. Since liver tumorigenesis is driven by apoptosis, we examined the effect of overt hepatocyte apoptosis in a mouse model of NASH using mice lacking myeloid cell leukemia 1 (Mcl1), a pro-survival member of the BCL-2 protein family. Hepatocyte-specific Mcl1 knockout (Mcl1^∆hep^) mice and control littermates were fed chow or FFC (high saturated fat, fructose, and cholesterol) diet, which induces NASH, for 4 and 10 months. Thereafter, liver injury, inflammation, fibrosis, and tumor development were evaluated biochemically and histologically. Mcl1^∆hep^ mice fed with the FFC diet for 4 months displayed a marked increase in liver injury, hepatocyte apoptosis, hepatocyte proliferation, macrophage-associated liver inflammation, and pericellular fibrosis in contrast to chow-fed Mcl1^∆hep^ and FFC diet-fed Mcl1-expressing littermates. After 10 months of feeding, 78% of FFC diet-fed Mcl1^∆hep^ mice developed liver tumors compared to 38% of chow-fed mice of the same genotype. Tumors in FFC diet-fed Mcl1^∆hep^ mice were characterized by cytologic atypia, altered liver architecture, immunopositivity for glutamine synthetase, and histologically qualified as HCC. In conclusion, this study provides evidence that excessive hepatocyte apoptosis exacerbates the NASH phenotype with enhancement of tumorigenesis in mice.

## Introduction

Obesity-related co-morbidities including non-alcoholic fatty liver disease (NAFLD) have paralleled the increasing prevalence of obesity and are a major public health concern. NAFLD is currently the most common chronic liver disease with an estimated worldwide prevalence of 25%^1^. NAFLD encompasses a spectrum of liver conditions ranging from steatosis to a more severe and progressive disease termed nonalcoholic steatohepatitis (NASH). NASH is characterized by hepatocyte injury and apoptosis, hepatic infiltration by inflammatory cells and varying degrees of fibrosis, and may culminate in liver cirrhosis and/or the development of hepatocellular carcinoma (HCC)^2^. Advanced fibrosis in particular has been reported to be associated with a significantly increased risk for HCC. For example, up to 13% of individuals with NASH-related cirrhosis develop HCC over 3 years in contrast to 0–3% of NAFLD cohort without cirrhosis followed for a period of 10–20 years^3^. Thus, cirrhosis is associated with an increased risk but at the same time is not required for HCC development in NAFLD. Congruently, up to 45–50% of patients with NAFLD-related HCC develop the malignancy in the absence of cirrhosis^3^. The mechanisms of non-cirrhotic carcinogenesis in NAFLD may or may not be different from that occurring in cirrhotic liver. Recently, we identified central players in the pathogenesis of NASH and NASH-driven liver cancer development at the cellular and molecular level^4,5^. Nevertheless, a better understanding of the molecular mechanisms underlying NAFLD-associated hepatocarcinogenesis is urgently needed.

Recent studies using various preclinical models have clearly demonstrated that excessive hepatocyte apoptosis can drive liver tumorigenesis^6^. This has been well established in genetic mouse models in the context of dysregulated expression of BCL-2 family proteins, which control the mitochondrial pathway of apoptosis. Liver-specific deletion of myeloid cell leukemia 1 (Mcl1), an anti-apoptotic protein, results in spontaneous hepatocyte apoptosis, compensatory proliferation and eventually HCC^7,8^. Interestingly, in this mouse model, hepatocytes display a high degree of genomic instability and HCC develops in the absence of overt inflammation^8^. Our recent study also demonstrated that the risk for HCC development tightly correlates with the magnitude of hepatocyte apoptosis in early life of these transgenic mice^9^. In addition, perturbation of extrinsic apoptotic pathway may also lead to increased hepatocyte apoptosis, compensatory proliferation and HCC. Compound deletion of proteins involved in the death receptor signaling cascade, receptor-interacting protein kinase 1 and TNF receptor-associated factor 2, in liver parenchymal cells causes excessive hepatocyte apoptosis, compensatory proliferation, and development of HCC^10^. Excessive hepatocyte apoptosis in these mice is likely due to simultaneous activation of caspase 8 and impaired activation of canonical NF-κB. In other mouse models, dysregulated physiological NF-κB activation in hepatocytes due to ablation of NF-κB essential modulator (NEMO) or transforming growth factor (TGF)-β-activated kinase 1 (TAK1) also results in excessive hepatocyte cell death and eventual hepatocarcinogenesis^11,12^. Altogether, these preclinical studies support the concept that aberrant hepatocyte apoptosis may drive liver carcinogenesis.

Hepatocyte apoptosis is significantly increased in NASH and correlates with disease severity^13^; however, whether the proapoptotic environment of fatty liver is tumor promoting has not been established. Therefore we asked: Is apoptosis a driving factor in tumorigenesis in fatty liver disease? To address this question we combined a model of diet-induced NASH with a liver apoptosis-prone model, i.e., hepatocyte-specific Mcl1 knockout mice. We observed that excessive hepatocyte apoptosis due to Mcl1 loss drives HCC development in fatty liver disease. Thus, this study provides evidence that excessive hepatocyte apoptosis is tumor promoting also in fatty liver disease and the anti-apoptotic protein Mcl1 is tumor suppressive in mouse liver affected by NASH.

## Materials and methods

### Animals and experimental design

All animal experiments conformed to the relevant regulatory standards and were approved by the Cantonal Veterinary Office (license ZH136/14). Conditional hepatocyte-specific Mcl1 knockout mice (homozygous Mcl1^flox/flox^-Albumin-Cre referred to as Mcl1^∆hep^) and control littermates (Mcl1^flox/flox^ referred to as WT) on C57BL/6 background were generated and genotyped as previously described^7^. Animals were housed under temperature-controlled conditions, humidity, and a 12 h/12 h light–dark circadian cycle. Mcl1^∆hep^ and WT mice used in the study were males, littermates and co-housed in the same cages. At two months of age, mice were placed on a diet high in saturated fat and cholesterol (40% calories from fat, 0.2% cholesterol, AIN-76A Western Diet, TestDiet, St. Louis, MO) with exogenous glucose plus fructose (42 g/L) added to the drinking water or maintained on a standard chow and water for 4 and 10 months. The former diet was termed the FFC (high saturated fat, high fructose, and high cholesterol) diet. The FFC diet feeding in mice results in the development of obesity, insulin resistance, adipose tissue inflammation, and NASH with hepatocyte ballooning and fibrosis, which displays high fidelity to human NASH^14–19^. Of note, mice had ad libitum access to food and water, and their food and water intake was not recorded. At the end of the feeding period, mice were sacrificed under general anesthesia, and blood and liver were harvested for further analysis. Investigators were not blinded to the group allocation during the experiment, data acquisition and analyses.

### Biochemical analysis

Serum AST and ALT activities were determined on a Roche Modular System (Roche Diagnostics) with a commercially available automated colorimetric system at the Institute of Clinical Chemistry, University Hospital Zurich, using a Hitachi P-Modul (Roche). Triglyceride concentrations in mouse liver homogenates were measured using EnzyChrome triglyceride assay kit (BioAssay Systems) as previously described by us^20^.

### Histopathology, immunohistochemistry, and Sirius red staining

For histological review, liver tissue was diced into sections, fixed in 4% paraformaldehyde, and then embedded in paraffin. Tissue sections (2 μm) were prepared using a microtome and placed on glass slides. Hematoxylin and eosin (H&E) staining was performed according to standard techniques. For immunohistochemistry, paraformaldehyde-fixed paraffin-embedded liver tissue sections were deparaffinized, and hydrated. Incubation in Ventana buffer and staining was performed on a NEXES immunohistochemistry robot (Ventana Instruments) using an IVIEW DAB Detection Kit (Ventana) or on a Bond MAX (Leica). Immunostainings were performed as described before^9^ with antibodies against the following proteins: Ki67, 1:200 dilution (Lab Vision, #RM-9106-S); cleaved caspase 3, 1:300 dilution (Cell Signaling Technology, #9661); F4/80, 1:50 dilution (BMA Biomedicals, #T-2006); glutamine synthetase, 1:800 dilution (Abcam, #ab16802); collagen IV, 1:50 dilution (Cedarlane, #CL50451AP); GP73, 1:100 dilution (Santa Cruz Biotechnology, #sc-48011); and β-catenin, 1:25 dilution (Cell Signaling Technology, #9582). The tissue sections were counterstained with hematoxylin. For virtual microscopy and archiving, histological and immunohistochemical images were digitalized using a Nano Zoomer C9600 Virtual Slide Light microscope scanner by Hamamatsu using NDP View Software. To quantify immunohistochemical staining for F4/80, five animals per group and at least five random pictures of liver (20×) tissue per animal were assessed by morphometry using color deconvolution and ImageJ software (NIH). To quantify immunohistochemical staining for cleaved caspase 3 and Ki67, positively stained hepatocytes were counted in 20 random microscopic fields (20×) in five animals per group. Liver fibrosis was assessed using Sirius red staining on paraformaldehyde-fixed, paraffin-embedded sections. Red-stained collagen fibers were quantified by digital image analysis using ImageJ in five animals per group. Sudan red staining (0.25% Sudan IV in ethanolic solution) was performed on frozen liver sections (5 μm) according to a standard method and slides were scanned with a Nano Zoomer (Hamamatsu, Japan).

### Quantitative real-time polymerase chain reaction (qPCR)

Total RNA from liver tissue or liver tumors was isolated with TRIZOL reagent (Invitrogen) and was reverse transcribed using iScript cDNA synthesis kit (Bio-Rad). Quantification of gene expression was performed by real-time polymerase chain reaction using SYBR green fluorescence on a LightCycler 480 instrument (Roche). Specific primers are listed in Supplemental Table 1. Target gene expression was calculated using ΔΔCt method. Expression was normalized to 18S expression levels, which were stable across all experimental groups. All data represent fold change over expression in Mcl1^flox/flox^ (WT) mice fed standard chow diet.

### Immunoblot analysis

Liver tissue (~20 g for FFC-fed mice, ~10 g for chow-fed mice) was homogenized in lysis buffer (T-PER reagent, Roche, #11,697,498,001, containing Halt Protease and Phosphatase Inhibitor Single-Use Cocktail, Thermo Fisher, #78442) followed by centrifugation at 15,000×*g* for 15 min at 4 °C to remove debris. Protein concentration was determined by the Bradford assay method. Equal amounts of protein were loaded onto SDS-PAGE gel, transferred to nitrocellulose membrane and incubated overnight with primary antibodies: Mcl1 (Rockland Inc., #600–401–394S, 1:2500 dilution) and GAPDH (Millipore, #3155980, 1:5000 dilution). Next day, membranes were washed, incubated with fluorochrome-conjugated secondary antibodies (IR Dye 800Rb, LI-COR, #926–32213; IR Dye 680Mo, LI-COR, #926–68072) and imaged using ChemiDoc MP Imaging System (Bio-Rad). GAPDH was used as a loading control. Densitometry-based quantification of the protein bands was performed using Image Lab software (Bio-Rad).

### Cytokine and chemokine protein array

Proteome Profiler Mouse Cytokine Array Kit (R&D Systems) was used to assess protein levels in mouse liver tissue. Liver tissue samples (~20 g for FFC-fed mice, ~10 g for chow-fed mice) were homogenized according to manufacturer’s instructions. Protein concentrations in liver lysates were measured and adjusted to equal levels. Four samples per group (representing four mice) were pooled for the experiment. Protein array membranes were incubated with liver lysates (200 µg of protein in 4 mL) overnight and detection of the signal was performed according to manufacturer’s instructions. Densitometry-based quantification was performed using Image Lab software (Bio-Rad).

### Statistical analysis

Data are expressed as means ± SEM. The number of mice used for analyses is listed in the figure legend. Four-months-long and 10-months-long mouse feeding studies were carried out once. Statistical methods were not applied to predetermine sample size; however, our animal sample size is similar to those reported in previous animal studies focused on NASH. No randomization method was used to determine how animals were allocated to experimental groups. No data were excluded from the study. Differences between multiple groups were analyzed by one-way analysis of variance (ANOVA). Individual group means were compared with Student’s unpaired *t*-test in which *p* < 0.05 was the minimum requirement for a statistically significant difference. Non-parametric data (NAS and tumor incidence) were analyzed using Mann–Whitney test. All analyses were performed using GraphPad Prism 8 software (San Diego, CA).

## Results

### Hepatocyte-specific Mcl1 deficiency accentuates liver injury in a murine model of diet-induced obesity with NASH

**Fig. 1:**
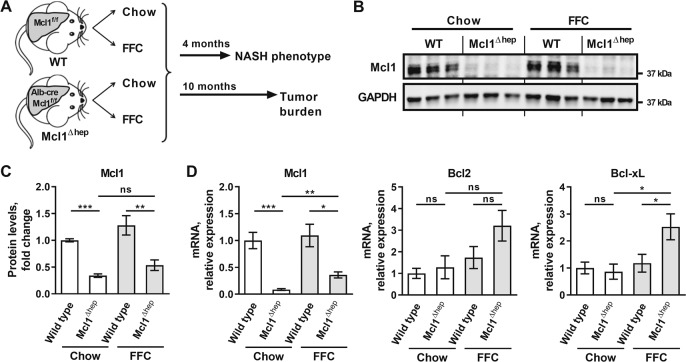
Study design. Mcl1^∆hep^ mice and control littermates (Mcl1^flox/flox^, referred to as WT) were placed on standard chow or diet high in fat, fructose and cholesterol (FFC diet). **a** One cohort of mice was sacrificed after 4 months of feeding to assess NASH phenotype. Another cohort of mice was sacrificed after 10 months of feeding to assess tumor development. **b** Immunoblot analysis of Mcl1 in whole liver tissue of mice fed for 4 months. GAPDH serves as a loading control. **c** Densitometry of the Mcl1 immunoblot in the liver tissue. Mcl1 protein levels were normalized to GAPDH levels. Chow-WT *n* = 6 mice; Chow-Mcl1^∆hep^ *n* = 5 mice; FFC-WT *n* = 6 mice; FFC-Mcl1^∆hep^ *n* = 6 mice. **d** mRNA expression of anti-apoptotic proteins in whole liver tissue of mice fed for 4 months. Chow-WT *n* = 8 mice; Chow-Mcl1^∆hep^ *n* = 5 mice; FFC-WT *n* = 12 mice; FFC-Mcl1^∆hep^ *n* = 7 mice; Bars represent mean ± SEM. ***p < 0.001, **p *<* 0.01, *p *<* 0.05 or not significant (ns).

To explore the role of hepatocyte apoptosis in obesity-induced NASH, hepatocyte-specific Mcl1 knockout mice (referred to as Mcl1^∆hep^) and control littermates (Mcl1^flox/flox^ referred to as WT) were placed on standard chow or high-fat, high-fructose, high-cholesterol (FFC) diet for 4 months (Fig. 1a). The FFC diet feeding is a well characterized NASH model with features of obesity, insulin resistance, adipose tissue inflammation, and steatohepatitis with hepatocyte ballooning and fibrosis, displaying high fidelity to human NASH^14,16,19,21–23^. To validate efficiency of cre recombinase-mediated deletion, we assessed Mcl1 at both mRNA and protein levels. Indeed, Mcl1 expression was diminished in the livers of Mcl1^∆hep^ mice (Fig. 1b–d). Hepatic expression of other anti-apoptotic proteins, Bcl2 and Bcl-xL, was not affected in chow-fed Mcl1^Δhep^ mice compared to control (WT) littermates (Fig. 1d), suggesting that the loss of Mcl1 did not cause compensatory expression of other pro-survival genes of Bcl2 family.

**Fig. 2:**
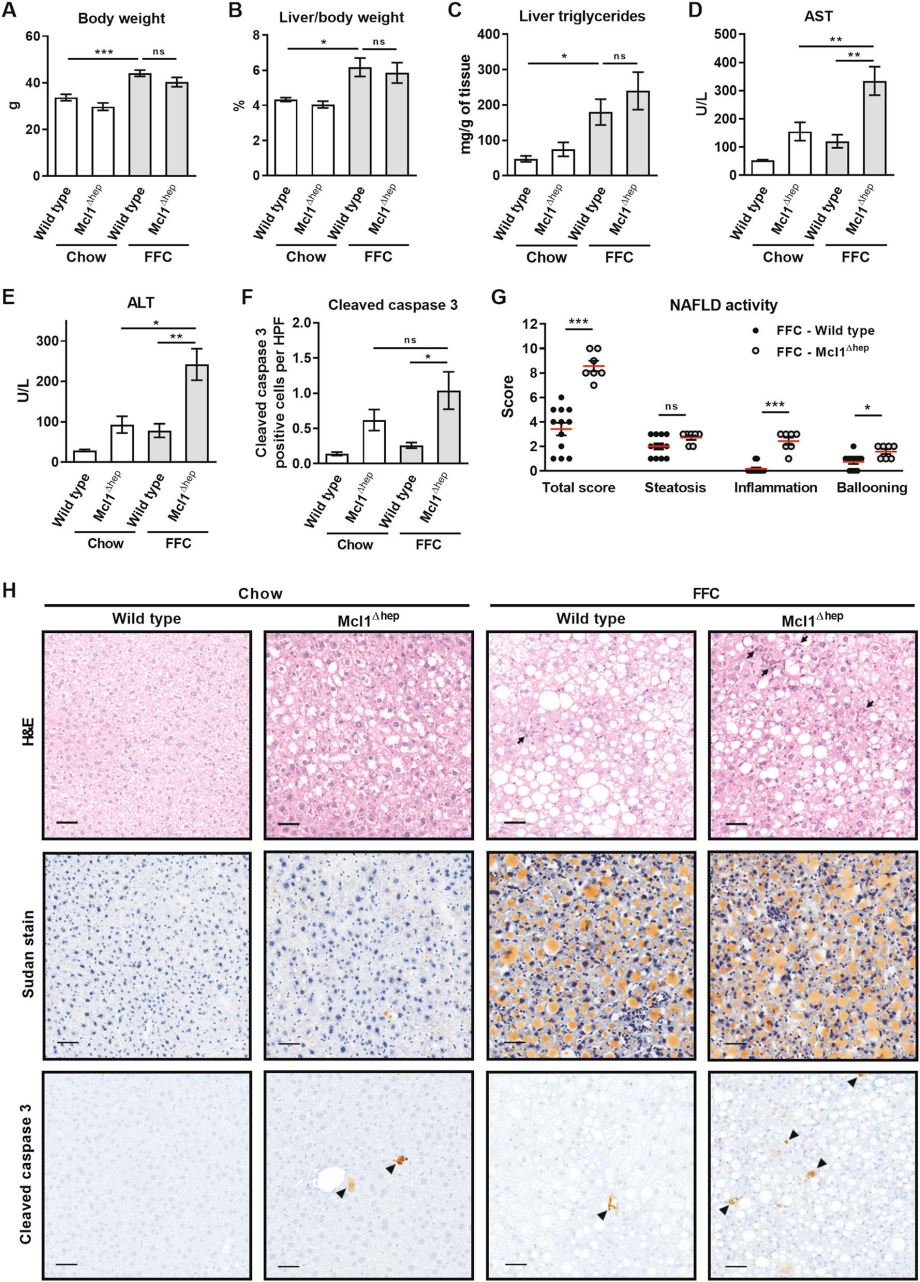
Mcl1 deficiency in hepatocytes exacerbates liver injury induced by the FFC diet. Mcl1^∆hep^ mice and control littermates (WT) were placed on standard chow or FFC diet for 4 months. Blood and livers were harvested at the end of the study. **a** Body weight at the end of the feeding study. **b** Liver weight as percentage of total body weight. **c** Liver triglycerides were measured in tissue homogenates using biochemical assay. **d**, **e** Liver injury was assessed by serum AST and ALT activity. **f** Hepatic apoptosis was assessed by immunohistochemistry and counting cleaved caspase 3-positive cells per 20× fields. *n* = 5 mice/group. **g** NAFLD activity score. FFC-WT *n* = 12 mice; FFC-Mcl1^∆hep^ *n* = 7 mice; **h** Representative images of H&E staining (arrows point to inflammatory foci), Sudan red staining for lipids, and immunohistochemistry for cleaved caspase 3 (arrowheads) in liver tissue samples (scale bar 50 μm). Bars represent mean ± SEM. **a**–**e** Chow-WT *n* = 9 mice; Chow-Mcl1^∆hep^ *n* = 5 mice; FFC-WT *n* = 12 mice; FFC-Mcl1^∆hep^ *n* = 7 mice; ***p < 0.001, **p < 0.01, *p < 0.05 or not significant (ns).

In the current study, both WT and Mcl1^∆hep^ mice on the FFC diet developed a similar degree of obesity, increased liver weight and steatosis compared to standard chow-fed mice (Fig. 2a–c). FFC-diet induced hepatic macrovesicular steatosis did not differ between WT and Mcl1^∆hep^ mice on the FFC diet, as assessed by histology, Sudan red stain and biochemical quantification of neutral triglycerides (Fig. 2c, h). Mcl1^∆hep^ mice on FFC diet, however, displayed increased liver injury as manifest by substantially elevated aspartate aminotransferase (AST) and alanine aminotransferase (ALT) values compared to WT FFC-fed animals (Fig. 2d, e). As hepatocyte apoptosis is a prominent histopathologic feature of NASH^13^, we next examined apoptosis in liver tissue samples using immunohistochemistry for cleaved caspase 3. As expected, WT mice on the FFC diet demonstrated a significant increase in liver cleaved caspase 3-positive cells compared to standard chow-fed animals (Fig. 2f, h). Consistent with the elevated serum AST and ALT levels, FFC-fed Mcl1^∆hep^ mice displayed a marked increase in cleaved caspase 3-positive cells compared to WT FFC-fed mice (Fig. 2f, h, arrowheads). Congruent with our prior studies, chow-fed Mcl1^∆hep^ mice also displayed spontaneous liver injury as evidenced by increased serum transaminases and increased number of cleaved caspase 3-positive cells in the liver^7,8^. Finally, liver histology of FFC-fed mice was assessed by a pathologist using human NAFLD activity score (NAS)^24^. Compared to FFC-fed WT mice, FFC-fed Mcl1^∆hep^ mice had a significantly higher NAS, which was largely due to increased lobular inflammation and ballooning (Fig. 2g). These data collectively suggest that Mcl1 deficiency in hepatocytes accentuates liver injury and NAFLD severity in FFC diet-fed mice.

### Hepatocyte-specific Mcl1 knockout mice fed the FFC diet display exacerbated macrophage-associated inflammation and fibrosis

**Fig. 3:**
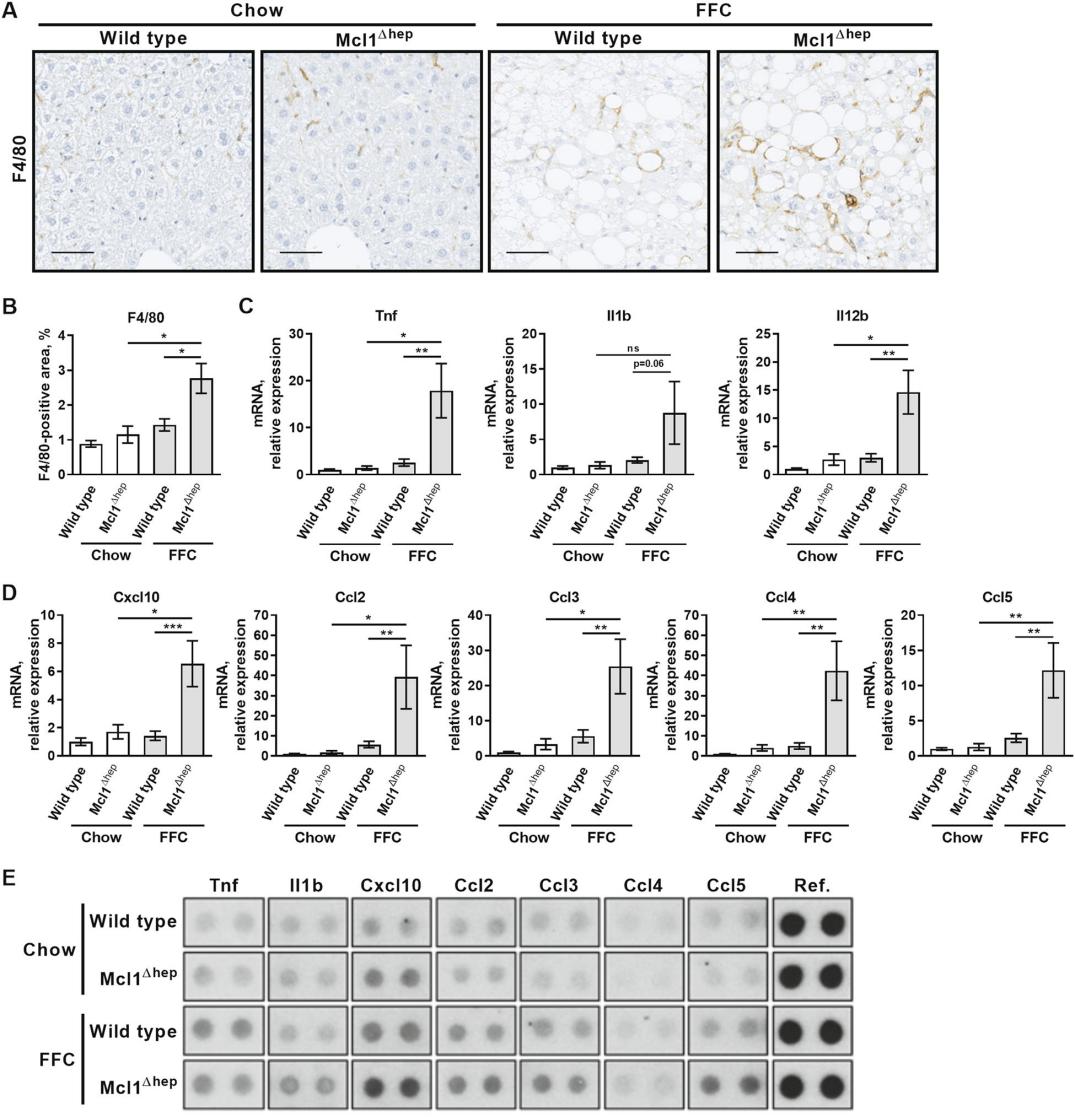
Mcl1 deficiency in hepatocytes accentuates inflammation in mice fed the FFC diet for 4 months. Mcl1^∆hep^ mice and control littermates (WT) were fed standard chow or FFC diet for 4 months. Livers were harvested at the end of the study for following analyses: **a** Representative images of immunohistochemistry for F4/80, a pan-macrophage marker (scale bar 50 μm). **b** Morphometric quantification of F4/80 immunohistochemistry. *n* = 5 mice/group. **c** Whole liver mRNA expression of proinflammatory cytokines by qPCR. **d** Whole liver mRNA expression of proinflammatory chemokines by qPCR. **e** Pooled liver lysate samples (four mice per group) were used for protein array. Bars represent mean ± SEM. **c**, **d** Chow-WT *n* = 8 mice; Chow-Mcl1^∆hep^ *n* = 5 mice; FFC-WT *n* = 12 mice; FFC-Mcl1^∆hep^ *n* = 7 mice; ***p < 0.001, **p < 0.01, *p < 0.05 or not significant (ns).

Inflammation in both murine and human NASH is characterized by accumulation and activation of proinflammatory macrophages^25,26^. Therefore, we explored the extent to which cells of this lineage accumulated in liver after 4 months of feeding. Using immunohistochemistry on liver tissue sections, we detected a substantial increase in F4/80-positive cells in the FFC diet-fed Mcl1^∆hep^ mice compared to FFC-fed WT mice, suggesting a marked accumulation of macrophages (Fig. 3a, b). Macrophage activation was examined by measuring mRNA expression of proinflammatory cytokines, tumor necrosis factor (Tnf), interleukin (Il) 1b and 12b, and chemokines, chemokine (C-X-C motif) ligand 10 (Cxcl10) and chemokine (C–C motif) ligands (Ccl), known to be secreted by activated macrophages. Indeed, hepatic mRNA levels of cytokines Tnf and Il12b and chemokines Cxcl10, Ccl2, Ccl3, Ccl4, and Ccl5 were significantly increased in FFC-fed Mcl1^∆hep^ mice compared to FFC-fed WT mice (Fig. 3c, d). In addition, we profiled 40 cytokines and chemokines in the liver tissue at a post-transcriptional level using a protein array (Suppl. Fig. 1). We found that hepatic protein levels of cytokines and chemokines showed similar trends as mRNA. Compared to other experimental groups, the FFC-fed Mcl1^∆hep^ mice had the highest levels of Il1b, Cxcl10, Ccl2, Ccl3, and Ccl5 (Fig. 3e; Suppl. Fig. 1). Thus, these observations extend the histologic assessment by NAS (Fig. 2g) and confirm that hepatocyte Mcl1 deficiency promotes FFC diet-induced hepatic accumulation and activation of macrophages in the FFC model of NASH.

**Fig. 4:**
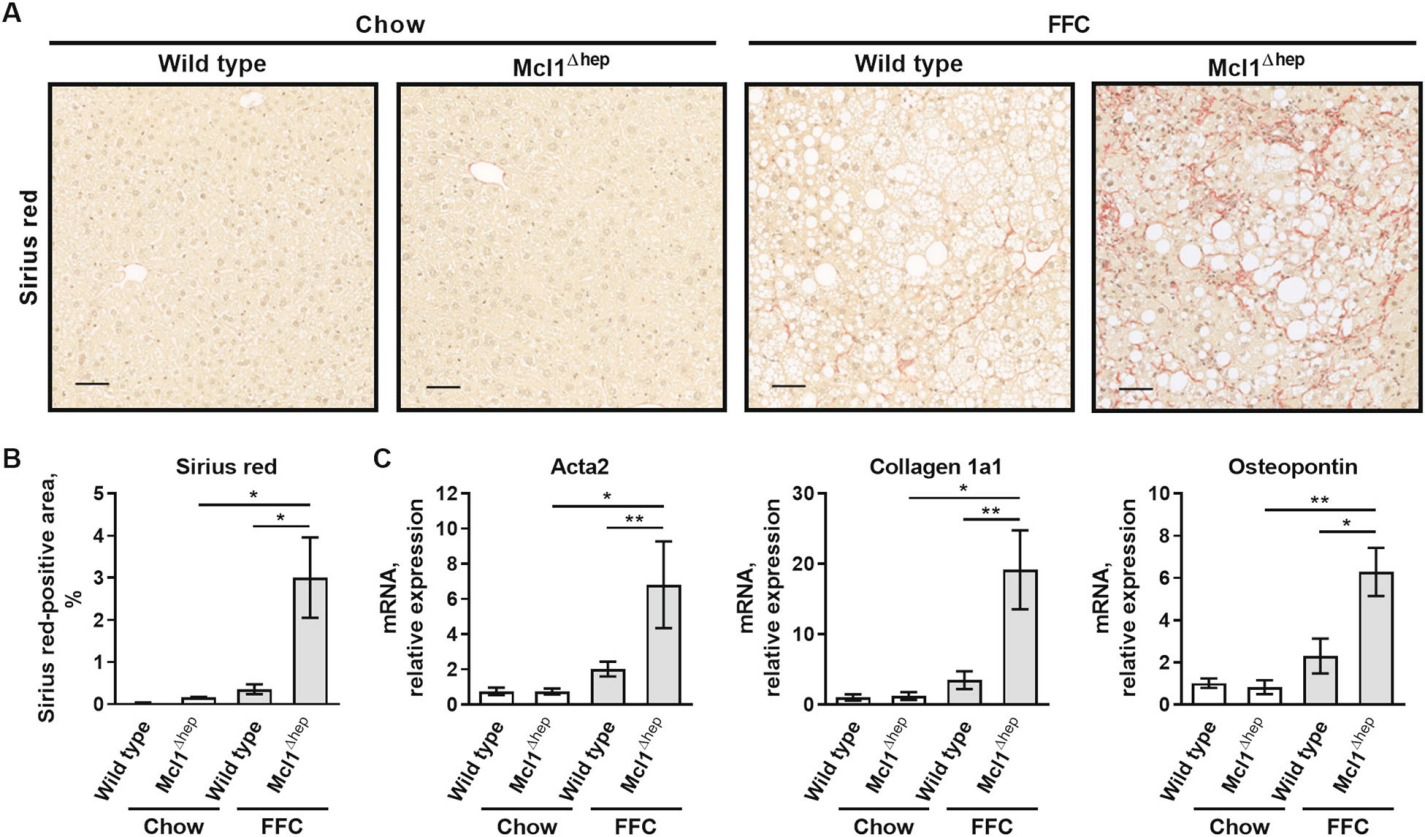
Mcl1 deficiency in hepatocytes accentuates fibrosis in mice fed the FFC diet for 4 months. Mcl1^∆hep^ mice and control littermates (WT) were fed standard chow or FFC diet for 4 months. Livers were harvested at the end of the study for following analyses: **a** Representative images of sirius red staining in the liver tissue (scale bar 50 μm). **b** Morphometric analysis of sirius red staining. *n* = 5 mice/group. **c** Whole liver mRNA expression of markers for hepatic stellate cell activation. Chow-WT *n* = 8 mice; Chow-Mcl1^∆hep^ *n* = 5 mice; FFC-WT *n* = 12 mice; FFC-Mcl1^∆hep^ *n* = 7 mice; Bars represent mean ± SEM. **p < 0.01, *p < 0.05.

We next examined the effect of hepatocyte Mcl1 deficiency on the FFC diet-induced liver fibrosis. Sirius red staining detecting collagen tissue deposition demonstrated the presence of chicken wire-like perisinusoidal fibrosis in the liver of FFC diet-fed WT mice, which was significantly increased in FFC-fed Mcl1^∆hep^ mice (Fig. 4a, b). Consistent with the histological findings, increased liver fibrogenesis in FFC-fed Mcl1^∆hep^ mice was evident through upregulated mRNA levels of collagen 1a1, alpha smooth muscle actin (Acta2) and osteopontin, markers of activated hepatic stellate cells (Fig. 4c). Thus, Mcl1 deficiency in hepatocytes promotes FFC-diet induced liver fibrogenesis, presumably by increasing liver injury and consequently liver inflammation, as commonly seen in chronic liver disease^27,28^. Of note, although it does not indicate a causal relationship, in our cohort of mice, we found that the degree of fibrosis strongly and positively correlated with the magnitude of hepatocyte injury (serum AST and ALT; Suppl. Fig. 2), suggesting that hepatocyte injury is linked to liver fibrogenesis.

### Hepatocyte-specific Mcl1 knockout mice fed the FFC diet exhibit increased hepatocyte proliferation

**Fig. 5:**
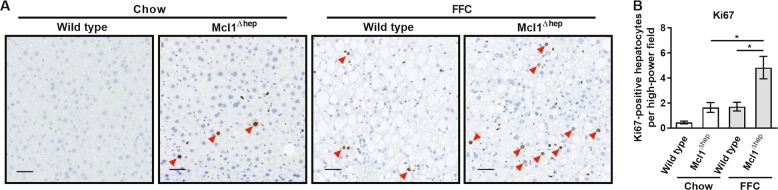
Mcl1 deficiency in hepatocytes increases hepatocyte proliferation in mice fed the FFC diet for 4 months. Mcl1^∆hep^ mice and control littermates (WT) were fed standard chow or FFC diet for 4 months. Livers were harvested at the end of the study to assess hepatocyte proliferation by immunohistochemistry for Ki67. **a** Representative images of immunohistochemistry for Ki67 (scale bar 50 μm). **b** Ki67-positive hepatocytes were counted in 20 random 20× fields. *n* = 5 mice/group; Bars represent mean ± SEM. *p < 0.05.

We have previously demonstrated that deletion of Mcl1 in hepatocytes results in greater hepatocyte proliferation in two-month-old mice fed standard chow diet^9^. Thus, we examined whether FFC diet-induced hepatocyte apoptosis was also linked to compensatory hepatocyte proliferation. Immunohistochemistry for Ki67 revealed that FFC-fed Mcl1^∆hep^ mice displayed a significant increase in number of Ki67-positive hepatocytes compared to both FFC-fed WT mice and chow-fed Mcl1^∆hep^ mice (Fig. 5a, b).

### Mcl1 deficiency in hepatocytes promotes tumor development in mice fed the FFC diet

**Fig. 6:**
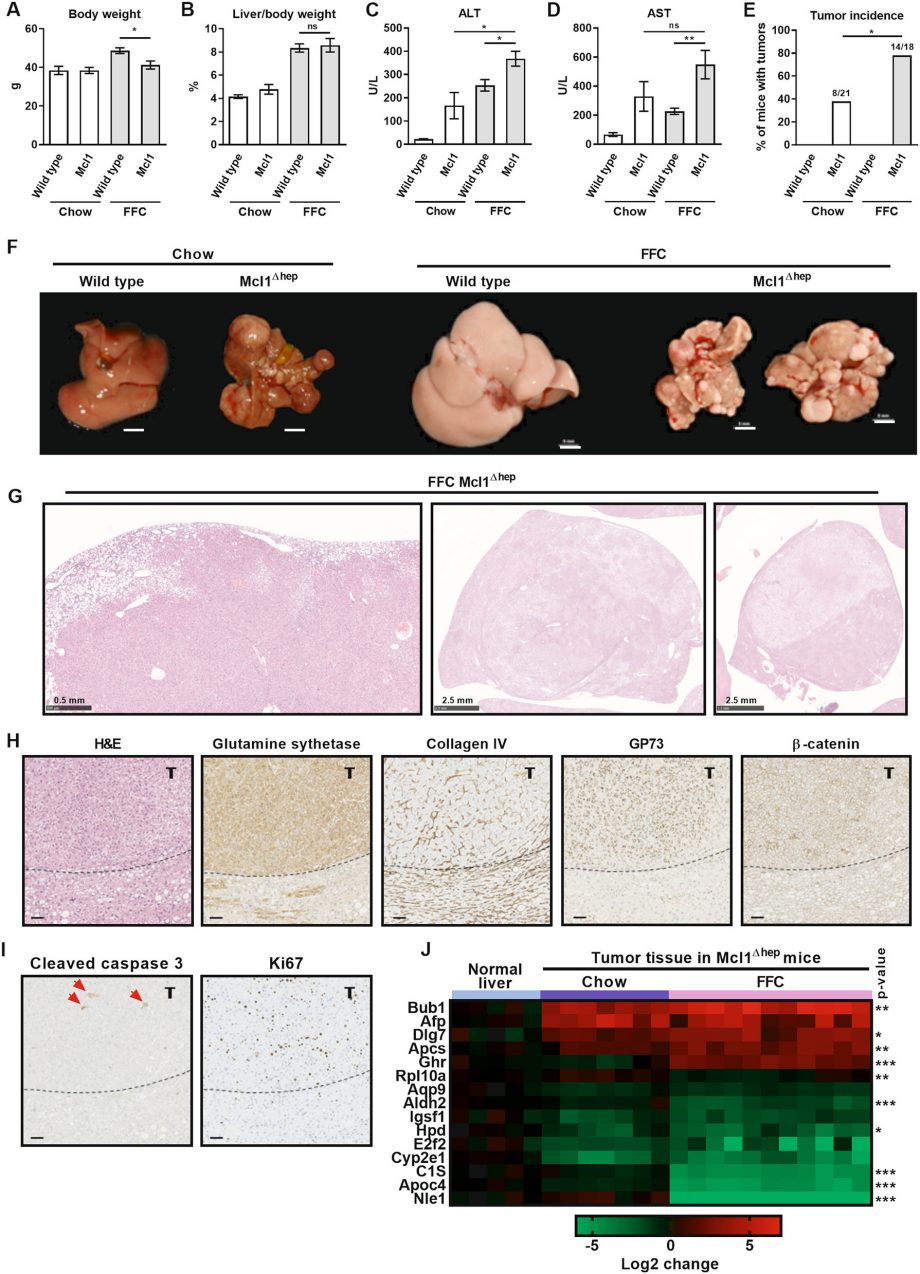
Mcl1 deficiency in hepatocytes promotes tumor development in mice fed the FFC diet for 10 months. Mcl1^∆hep^ mice and control littermates (WT) were placed on standard chow or FFC diet for 10 months. Blood and livers were harvested at the end of the study. **a** Body weight at the end of the feeding study. **b** Liver weight as percentage of total body weight. **c**, **d** Liver injury was assessed by serum AST and ALT activity. **e** Presence of tumors was assesses by histology. Chow-WT *n* = 9 mice; Chow-Mcl1^∆hep^ *n* = 21 mice; FFC-WT *n* = 14 mice; FFC-Mcl1^∆hep^ *n* = 18 mice. **f** Macroscopic view of livers (scale bar 5 mm). **g** Representative images of H&E-stained liver sections of FFC-fed Mcl1^∆hep^ mice. Left and middle image: tumors lack distinct borders; middle and right image: tumors show nodule-in-nodule appearance (scale bar 0.5 mm or 2.5 mm as indicated). **h**, **i** Liver sections of FFC-fed Mcl1^∆hep^ mice were stained for H&E, glutamine synthetase, collagen IV, GP73, β-catenin, cleaved caspase 3 (red arrows) and Ki67 and representative images are shown. Dotted line marks tumor (t) border (scale bar 50 μm). **j** Real-time qPCR analysis of genes known to be dysregulated in liver cancer. *P* value calculated for differences found between tumors of Mcl1^∆hep^ mice fed chow vs FFC diet. Bars represent mean ± SEM. **a**, **b** Chow-WT *n* = 5 mice; Chow-Mcl1^∆hep^ *n* = 13 mice; FFC-WT *n* = 14 mice; FFC-Mcl1^∆hep^ *n* = 18 mice; **c**, **d** Chow-WT *n* = 5 mice; Chow-Mcl1^∆hep^ *n* = 10 mice; FFC-WT *n* = 12 mice; FFC-Mcl1^∆hep^ *n* = 13 mice; **p *<* 0.01, *p < 0.05 or not significant (ns).

Another cohort of WT and Mcl1^∆hep^ mice were fed a standard chow or the FFC diet for 10 months (Fig. 1a). At the time of sacrifice, FFC-fed Mcl1^∆hep^ mice had significantly lower body weight compared to FFC-fed WT mice (Fig. 6a), but the liver to body weight ratio was not different (Fig. 6b). Similar to the 4-month feeding, FFC-fed Mcl1^∆hep^ mice had significantly increased serum transaminases, ALT and AST, compared to FFC-fed WT mice (Fig. 6c, d). Macroscopic examination of the livers revealed that 8 of 21 chow-fed Mcl1^∆hep^ mice were bearing tumors (incidence ~38%, Fig. 6e, f). The tumors in chow-fed Mcl1^∆hep^ mice showed similar characteristics as previously reported by us in detail^8^. Advanced tumors histologically qualified as HCC. Most strikingly, 14 of 18 FFC-fed Mcl1^∆hep^ mice developed macroscopic liver tumors (incidence ~78%, Fig. 6e, f), confirmed by histologic analyses. Interestingly, tumor lesions in FFC-fed Mcl1^∆hep^ mice differed from tumors in chow-fed Mcl1^∆hep^ mice^8,29^ in respect to their growth pattern which frequently lacked distinct borders (Fig. 6g, left and middle image) but often showed nodule-in-nodule appearance (Fig. 6g, middle and right image). The tumors in FFC-fed Mcl1^∆hep^ mice displayed cellular atypia, altered liver-architecture with broadening of liver cell cords highlighted by collagen IV staining, and frequent immunoreactivity for glutamine synthetase, nuclear β-catenin and GP73 (Fig. 6h). Again, advanced tumors histologically qualified as HCC. In addition, these tumors displayed increased positivity for cleaved caspase 3 and were highly proliferative as indicated by increased immunoreactivity for Ki67 (Fig. 6i). To gain insight into tumor gene expression patterns, using qPCR we profiled candidate genes identified as being dysregulated in liver cancer^30^. We found several significant differences in tumor gene expression between chow and FFC diet (Fig. 6j). For example, the FFC diet feeding upregulated proliferation-related genes (e.g., spindle checkpoint genes Bub1 and Dlg7) in the liver tumors of Mcl1^∆hep^ mice. On the other hand, FFC diet feeding was associated with a downregulation of hepatocyte markers Aldh2 and Apoc4. These results suggest that the FFC diet feeding significantly affects both histologic features and transcriptome of liver tumors. Altogether, these data indicate that combination of aberrant hepatocyte apoptosis and fatty liver condition promotes hepatocarcinogenesis and changes the quality of tumors.

## Discussion

The present study tests the hypothesis that excessive hepatocyte apoptosis in fatty liver disease promotes liver tumorigenesis. The principal findings of this study indicate that in mice fed a NASH-inducing FFC diet, hepatocyte Mcl1 deficiency: (i) exacerbates liver injury, inflammation and fibrosis; (ii) further increases compensatory hepatocyte proliferation; and (iii) promotes HCC development. These findings are discussed in detail below.

To study NASH in vivo, we utilized a well-established diet-induced mouse model of NASH^14,22^. This model includes a diet high in saturated fat, cholesterol, and addition of high-fructose syrup in the drinking water (thus termed FFC diet) and was developed to replicate the western fast food diet. This model displays a high fidelity to the metabolic profile observed in humans with NASH, including obesity, hyperlipidemia, and insulin resistance. Importantly, FFC diet-induced NASH recapitulates key features of human disease including hepatocyte neutral lipid accumulation, hepatocyte injury and cell death, the presence of ballooned hepatocytes, hepatic infiltration of inflammatory cells, and liver fibrosis^14,22^. Similar to other dietary NASH models, FFC-fed C57BL/6 mice do not develop NASH-related HCC within the time frame of 12 months^22^.

Hepatocyte survival and cell death are tightly regulated by proteins belonging to BCL-2 family. In particular, Mcl1 has been previously demonstrated to play a crucial role in the regulation of hepatocyte survival and cell death. Hepatocyte-specific deletion of Mcl1 in vivo leads to spontaneous hepatocyte apoptosis and compensatory proliferation, both of which can be detected in 1-month-old to 4-month-old Mcl1^∆hep^ mice^7^. Excessive hepatocyte apoptosis is also a pathologic hallmark of NASH and magnitude of hepatocyte apoptosis correlates with NASH severity, in particular with the degree of inflammatory activity and fibrosis^13^. In the present study, Mcl1^∆hep^ mice placed on the NASH-inducing FFC diet for 4 months displayed severe liver injury and excessive hepatocyte apoptosis. These data underscore that Mcl1 is a critical antiapoptotic factor in hepatocytes not only under steady-state conditions but especially when liver is challenged with an additional proapoptotic insult such as western-like diet. Our previous studies uncovered that hepatocyte cell death in the FFC diet NASH model is mediated, at least in part, via signaling cascade initiated by TNF-related apoptosis-inducing ligand (TRAIL) receptor as FFC-fed mice lacking TRAIL, TRAIL receptor or caspase 8 (in hepatocytes) display attenuated liver injury and hepatocyte apoptosis^16,31,32^. In hepatocytes, death receptor-activated proapoptotic signaling converges on mitochondria-mediated cell death pathway^33^. Therefore, loss of mitochondrial antiapoptotic proteins, such as Mcl1, would be anticipated to further facilitate apoptosis as seen in FFC-fed Mcl1^∆hep^ mice. Moreover, liver expression of TRAIL and TRAIL receptor is upregulated in Mcl1^∆hep^ mice^9^, which may also accentuate FFC-induced hepatocyte apoptosis in the absence of hepatocyte Mcl1. Along with Mcl1, Bcl-xL is another antiapoptotic Bcl-2 family member maintaining hepatocyte homeostasis and preventing spontaneous apoptosis^34,35^. However, it is currently unknown whether deletion of Bcl-xL in hepatocytes would affect progression of fatty liver disease.

Whereas chow-fed Mcl1^∆hep^ mice were not particularly fibrosis-prone, Mcl1^∆hep^ mice fed the FFC diet for 4 months displayed marked liver fibrosis. Albeit multiple mechanisms may contribute to this observation, it is likely that increased liver fibrogenesis was a sequela of exacerbated liver injury and inflammation in FFC-fed Mcl1^∆hep^ mice. Consistent with our prior studies^7,8^, chow-fed Mcl1^∆hep^ mice did not develop overt inflammation at a cellular level. On the other hand, the FFC diet elicits liver inflammation mediated primarily by recruitment and activation of monocyte-derived macrophages^16,22,23^. It is likely that both infiltrating monocyte-derived macrophages and hepatic stellate cells contribute to liver fibrogenesis initiated by hepatocyte cell death. For example, we have previously reported that the engulfment of hepatocyte apoptotic bodies by stellate cells is profibrogenic^36^. In addition, stressed and apoptotic hepatocytes also release chemokines (e.g., CCL2, CXCL10), which serve as chemoattractants for monocyte-derived macrophage recruitment to the liver^37–40^. These liver recruited macrophages are considered proinflammatory and profibrogenic^13^. In the current study, hepatocyte Mcl1 deficiency in fatty liver markedly amplified macrophage-associated hepatic inflammation, which likely, along with hepatocyte cell death, contributed to increased hepatic stellate cell activation and fibrogenesis in FFC-fed Mcl1^∆hep^ mice.

Excessive apoptosis may induce compensatory proliferation, which in turn can promote tumorigenesis^41^. Indeed, Mcl1^∆hep^ mice displayed not only increased apoptosis but also increased hepatocyte proliferation, and this was substantially increased by the FFC diet feeding for 4 months. Indeed, after 10 months of the FFC diet feeding, we were able to detect tumor formation. We have previously observed that spontaneous chronic liver damage in Mcl1^∆hep^ results in HCC development in about 50% of 12-month-old mice^8^. In the present study, the tumor incidence in chow-fed Mcl1^∆hep^ mice was 38%. Strikingly, when Mcl1^∆hep^ mice were fed the FFC diet for 10 month the tumor incidence increased to 78%, while Mcl1-expressing (WT) mice on FFC diet did not develop any liver tumors. Similar to the FFC diet, other NASH models based on western-like diets in C57BL/6 mouse strain do not develop HCC in time period shorter than 12 months unless an additional insult is included. This additional insult can be a concurrent administration of hepatotoxic chemicals such as carbon tetrachloride, streptozotocin, or diethylnitrosamine^42,43^. Altogether, these findings suggest that fatty liver condition markedly promoted tumorigenesis in HCC susceptible liver.

We have observed qualitative differences between HCC formed in chow-fed vs. FFC-fed Mcl1^∆hep^ mice. The tumors in FFC-fed Mcl1^∆hep^ mice displayed a distinct growth pattern which compared to chow-fed Mcl1^∆hep^ mice^8,9^ more frequently lacked defined borders, but more often showed nodule-in-nodule appearance. It is conceivable that a proinflammatory microenvironment in NASH liver plays a role in this distinct tumor pattern. The FFC-fed Mcl1 knockout mice presented here also suggest that factors that are hepatotoxic have additive effects. This provides a useful model in which we can investigate the additive effects of hepatotoxic factors in a controlled manner, more closely simulating the clinical reality than models built on individual causes of liver disease (e.g., NAFLD and simultaneous chronic viral hepatitis). The concordance of the mouse model and the human disease may increase the chance of obtaining meaningful results^29^, such that FFC-fed Mcl1^∆hep^ mice may provide a more suitable preclinical model for a particular subtype of human liver cancer compared to other diet-based^4,5^ or genetically modified mouse models^8,9^. However, future mechanistic studies are warranted to fully uncover the cellular and molecular mechanism behind this observation.

In summary, the current study provides novel evidence that excessive hepatocyte apoptosis is a driving factor in liver tumorigenesis. In particular, our findings extend prior observations by demonstrating a tumor promoting effect of hepatocyte apoptosis in the setting of fatty liver disease. Our unique observations include an effect of hepatocyte Mcl1 deficiency on liver injury, macrophage-associated inflammation, fibrosis, and hepatocarcinogenesis. We propose that proapoptotic microenvironment in NASH promotes hepatocarcinogenesis and that the anti-apoptotic protein Mcl1 serves as a tumor suppressor in fatty liver.

## Supplementary information

Suppl. Figure Legends

Suppl. Table 1

Suppl. Figure 1

Suppl. Figure 2
