# Supplementary material for: Hepatocyte apoptosis is tumor promoting in murine nonalcoholic steatohepatitis
Source: Cell Death Dis. 2020 Feb 3;11(2):80. doi: 10.1038/s41419-020-2283-9 (PMC6997423; doi:10.1038/s41419-020-2283-9)
Supplement: Supplementary file 1 — Suppl. Figure Legends [file 41419_2020_2283_MOESM1_ESM.docx]

**SUPPLEMENTARY FIGURE LEGENDS**

**Suppl. Fig. 1. Hepatocyte Mcl1 deficiency in FFC-fed mice is associated with increased hepatic cytokines and chemokines levels.** Mcl1^∆hep^ mice and control littermates (WT) were fed standard chow or FFC diet for 4 months. Liver sample lysates were used for incubation with protein array membranes (pooled samples, 4 mice per group). (A) A representative image of protein array membranes; (B-E) Protein quantification by densitometry normalized to reference spots. Selected markers in (B) correspond to Fig. 3C-E. n=1

**Suppl. Fig. 2. Correlation between liver injury and liver fibrosis.** Mcl1^∆hep^ mice and control littermates (WT) were fed standard chow or FFC diet for 4 months. Serum and livers were harvested at the end of the study. Pearson correlation coefficient to measure the strength of an association between liver injury (serum AST or ALT values) and liver fibrosis (sirius red-stained area) was calculated using GraphPad Prism 8.
