# Supplementary material for: Hepatocyte apoptosis is tumor promoting in murine nonalcoholic steatohepatitis
Source: Cell Death Dis. 2020 Feb 3;11(2):80. doi: 10.1038/s41419-020-2283-9 (PMC6997423; doi:10.1038/s41419-020-2283-9)
Supplement: Supplementary file 2 — Suppl. Table 1 [file 41419_2020_2283_MOESM2_ESM.docx]

**Supplementary Table 1.** Primer sequences for quantitative real-time PCR in mouse tissues.

| Gene | Forward primer sequence (5’-3’) | Reverse primer sequence (5’-3’) |
| --- | --- | --- |
| Acta2 | GTC CCA GAC ATC AGG GAG TAA | TCG GAT ACT TCA GCG TCA GGA |
| Afp | GCC TGA ACTGACAGAGGAGCA | TTT AAA CGC CCA AAG CAT CAC |
| Aldh2 | AGG GAG CTG GGC GAG TAT G | TGT GTG GCG GTT TTT CTC AGT |
| Apcs | TGT TTG TCT TCA CCA GCC TTC TT | CGG AAA CACA GTG TAA AAT TCT GC |
| Apoc4 | AGC CAC TGG TGA CCA GAA CC | AGG AGG TGG TCT CTG GAG CTC |
| Aqp9 | CCC AGG CTC TTC ACT GCT CT | GGT TCG AGT GAT GCA TTT GGA |
| Bcl2 | ATG CCT TTG TGG AAC TAT ATG GC | GGT ATG CAC CCA GAG TGA TGC |
| Bcl-xL | TTC GGG ATG GAG TAA ACT GGG | CTC CTT GTC TAC GCT TTC CAC |
| Bub1 | GAT TGA TTA CTT TGG AGT TGC TGC | CAT GAT GTG AAA AAA TTC CTC CC |
| C1s | ATG GGA GAT GGG TAA ATG ACC A | TTA AAG AAG ACT TGC CAG GGA AA |
| Ccl2 | TTA AAA ACC TGG ATC GGA ACCA | GCA TTA GCT TCA GAT TTA CGG G |
| Ccl3 | TTC TCT GTA CCA TGA CAC TCT GC | CGT GGA ATC TTC CGG CTG TAG |
| Ccl4 | TTC CTG CTG TTT CTC TTA CAC CT | CTG TCT GCC TCT TTT GGT CAG |
| Ccl5 | GCT GCT TTG CCT ACC TCT CC | TCG AGT GAC AAA CAC GAC TGC |
| Collagen 1a1 | GCT CCT CTT AGG GGC CAC T | CCA CGT CTC ACC ATT GGG G |
| Cyp2e1 | TTT CTG CAG GAA AGC GCG | CTG CCA AAG CCA ATT GTA ACA G |
| Cxcl10 | CCA AGT GCT GCC GTC ATT TTC | GGC TCG CAG GGA TGA TTT CAA |
| Dlgap5 | GTG TCA CGT TTT GCC AGT CG | TCT GTT TCG CTC ATA CAC CCT |
| Dusp9 | CAA TGT CAC CCC CAA CCT TC | ACA GTT CTG CGA CAA GGC CT |
| E2f5 | ACC ATG GCT GCT CAA AAC CT | GCC GTA AAA GAG GAA ACA CAT CAG |
| Ghr | GCA GAT GTT CTG AAG GGA TGG | TCA CCC GCA CTT CAT GTT CTT |
| Hpd | GCC CAC ACT CTT CCT GGA AG | CAT TCC AGA CCT CAC ACC ATT G |
| Igsf1 | GGA AGG AGA AAG GCT GGT CAA | CCA AAT CCT GGA GCC ATC C |
| Il1b | GCA ACT GTT CCT GAA CTC AAC T | ATC TTT TGG GGT CCG TCA ACT |
| Il12b | TGG TTT GCC ATC GTT TTG CTG | ACA GGT GAG GTT CAC TGT TTC T |
| Mcl1 | GGT ATT TAA GCT AGG GTC ATT TGAA | TGC AGC CCT GAC TAA AGG TC |
| Nle1 | GCT GAA GGT GTG GGA TGT GA | GAG TCA TCT TCT CCA TAT CCG GA |
| Osteopontin | CTC CAT CGT CAT CAT CAT CG | TGC ACC CAG ATC CTA TAG CC |
| Rpl10a | GGC CTA AAC AAG GCT GGC A | CAT CGG TCA TCT TCA CGT GG |
| Tnf | CCC TCA CAC TCA GAT CAT CTT CT | GCT ACG ACG TGG GCT ACA G |
| 18s | CGC TTC CTT ACC TGG TTG AT | GAG CGA CCA AAG GAA CCA TA |
