# Supplementary figures and images for: Hepatocyte apoptosis is tumor promoting in murine nonalcoholic steatohepatitis
Source: Cell Death Dis. 2020 Feb 3;11(2):80. doi: 10.1038/s41419-020-2283-9 (PMC6997423; doi:10.1038/s41419-020-2283-9)

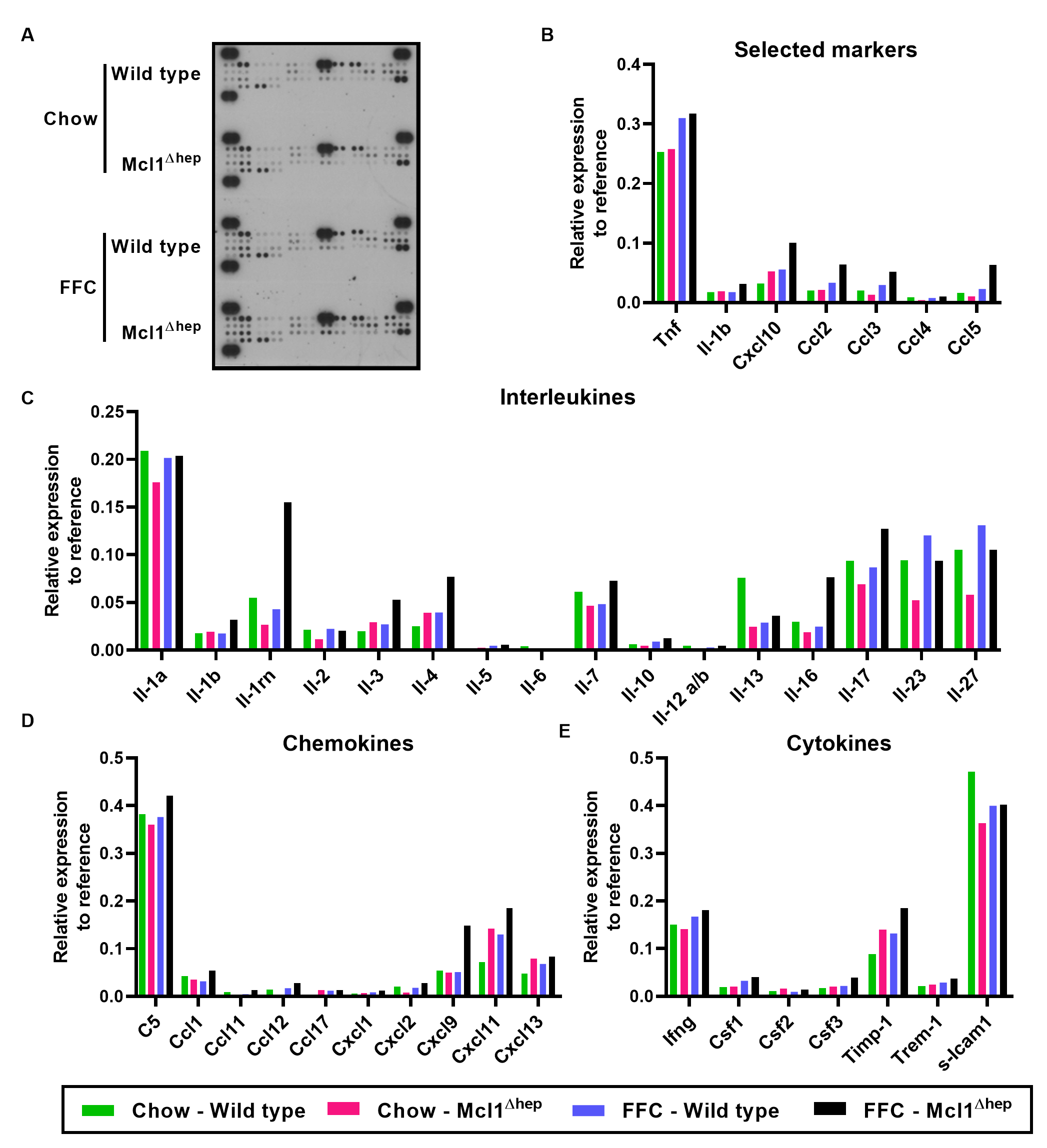

Supplement: Supplementary file 3 — Suppl. Figure 1 [file 41419_2020_2283_MOESM3_ESM.tif]
